# Plasma Glutaminyl-Peptide Cyclotransferase Mediates Glucosamine-Metabolism-Driven Protection Against Hypertension: A Mendelian Randomization Study

**DOI:** 10.3390/ijms252212106

**Published:** 2024-11-11

**Authors:** Fei Ge, Yu Sun, Cong-Cong Han, Zi-Liang Wei, Xin Guan, Si-Wan Guo, Shui Quan, Jia-Guo Zhou, Rui-Ping Pang

**Affiliations:** 1 Department of Pharmacology, Cardiac and Cerebrovascular Research Center, Zhongshan School of Medicine, Sun Yat-Sen University, Guangzhou 510275, China; 2 Guangdong Province Key Laboratory of Brain Function and Disease, Zhongshan School of Medicine, Sun Yat-Sen University, Guangzhou 510275, China; 3 Department of Physiology, Pain Research Center, Zhongshan School of Medicine, Sun Yat-Sen University, Guangzhou 510275, China

**Keywords:** mediation mendelian randomization, hypertension, glucosamine metabolism, glutaminyl-peptide cyclotransferase, glucosamine 6-phosphate N-acetyltransferase

## Abstract

Hypertension is one of the major risk factors for morbidity and mortality worldwide. In this study, Mendelian randomization was utilized to investigate how dietary supplement intake can impact hypertension based on circulating plasma metabolite genome-wide association study (GWAS) datasets, protein quantitative trait loci (pQTLs) of plasma proteins, and multiple public summary-level GWAS data. Pathway enrichment analysis combined with the results of inverse variance weighted Mendelian randomization revealed that a lower risk of hypertension was associated with the dietary intake of glucosamine, an anti-inflammatory supplement: odds ratio (OR) (95% CI): 0.888 (0.824–0.958). Additionally, glucosamine 6-phosphate N-acetyltransferase was identified as a protective factor against hypertension, OR (95% CI): 0.995 (0.992–0.998), shedding light on the potential protective mechanism of glucosamine. Mediation Mendelian randomization indicated that the protective effect of glucosamine metabolism was mediated by glutaminyl-peptide cyclotransferase, with a mediation proportion of 12.1% (5.9–18.2%), *p* < 0.05. This study offers new insights into preventive strategies for individuals with hypertension risk.

## 1. Introduction

Approximately 30% of adults worldwide are experiencing arterial hypertension [1]. High blood pressure has caused 56.1% more deaths over the past decade [2]. Hypertension is the most common preventable risk factor for cardiovascular diseases (CVDs), chronic kidney disease, and cognitive impairment [3]. It is also the leading single factor for global all-cause mortality and disability. One of the key causes is that modern dietary patterns are gradually transforming into a low “nutrient-dense” dietary pattern, characterized by high fat, excessive sugar and sodium, and inadequate fresh fruits or vegetables, increasing the severe risk of hypertension [4,5]. To reduce the risk of hypertension, healthy dietary patterns that increase protective ingredients such as the Dietary Approaches to Stop Hypertension (DASH) diet and the Mediterranean diet (MedDiet) have been recommended [6]. However, since changing dietary patterns can be challenging for most people to maintain, dietary supplements have been proposed as another acceptable protective measure. Therefore, it is important to investigate the association between dietary supplements and hypertension and their protective mechanisms. During an assessment conducted by the UK Biobank, participants filled out a questionnaire in which they were asked about their dietary intake of glucosamine, calcium, fish oil, iron, selenium, and zinc [7]. This work discusses the potential causal evidence of each dietary supplement on hypertension, where the intake of glucosamine was found to have a significantly negative association with hypertension.

Traditionally, glucosamine was recognized as a nonvitamin, nonmineral supplement for relieving osteoarthritis or joint pain [8,9]. Glucosamine has been permitted as a nutritional supplement or medication in nations such as the USA, Australia, and Ireland [7,10,11,12]. The widespread usage of glucosamine has also sparked discussions on whether it has a protective effect in other cases [13,14,15]. The anti-inflammatory effect of glucosamine has been gradually elucidated [8,13,14]. These pieces of evidence have led to more studies on the preventive effect of glucosamine on cancer [16,17], diabetes [18], CVDs [14,19,20], and all-cause death [21,22]. King et al. [19] claimed that the regular consumption of glucosamine is associated with lower CVD mortality in a US National Health and Nutrition Examination Survey cohort. Ma et al. [20] found that glucosamine supplementation was associated with a significantly lower risk of coronary CVDs in a 7-year follow-up study in the UK. Although excessive vascular O-GlcNAc levels can increase vascular reactivity, Xing et al. [23] found that chronic glucosamine treatment can reduce neointimal formation in injured arteries compared with vehicle treatment. Based on this work, Yao et al. [24] confirmed that the O-GlcNAcylation of TNFAIP3 protects against inflammation-induced vascular injury. Therefore, glucosamine supplementation has shown promise as a preventive approach for CVDs. However, the mechanism behind the negative association between glucosamine intake and hypertension has not been fully elucidated.

Studies have increasingly proven that metabolic alterations occur in hypertension, where multiple proteins and metabolites are biomarkers of hypertension [25,26,27]. Integrated proteomic and metabolomic analyses can elucidate the pathogenesis of diseases by tracing the characteristics of biomolecules [28]. We hope to uncover biomolecules with a causal association with both glucosamine metabolism and hypertension as potential mediators of the glucosamine-metabolism-driven protection against hypertension [29]. Mendelian randomization (MR) uses single-nucleotide polymorphisms (SNPs) as instrumental variables to examine causal associations [29]. SNPs are nonmodifiable, indicating the assignment of alleles to individuals before any exposure or outcome [30]. Therefore, confounding factors and reverse causation concerns are addressed using SNPs as instrumental variables. Over the last decade, genome-wide association studies (GWASs) have been published, allowing MR studies to explore the potential causal effects of different exposures and outcomes without the need to recruit more volunteers [31]. In this study, two-sample MR studies and a mediation MR analysis were employed to investigate the role of glucosamine metabolism in hypertension, utilizing GWAS datasets, protein quantitative trait locus (pQTL) datasets, and cis-expression quantitative trait locus (cis-eQTL) datasets. Firstly, the causal relationship between dietary glucosamine metabolism and hypertension was clarified after determining the causal relationship between glucosamine intake and hypertension. Secondly, circulating plasma proteins and metabolites were used in a two-step mediation MR analysis to identify a potential mediating pathway from glucosamine metabolism to hypertension via circulating biomolecules. This study aimed to elucidate the mechanisms underlying the protective role of glucosamine in preventing hypertension. We hope that this research will provide new insights for the prevention and treatment of hypertension.

## 2. Results

### 2.1. Study Design

**Figure 1:**
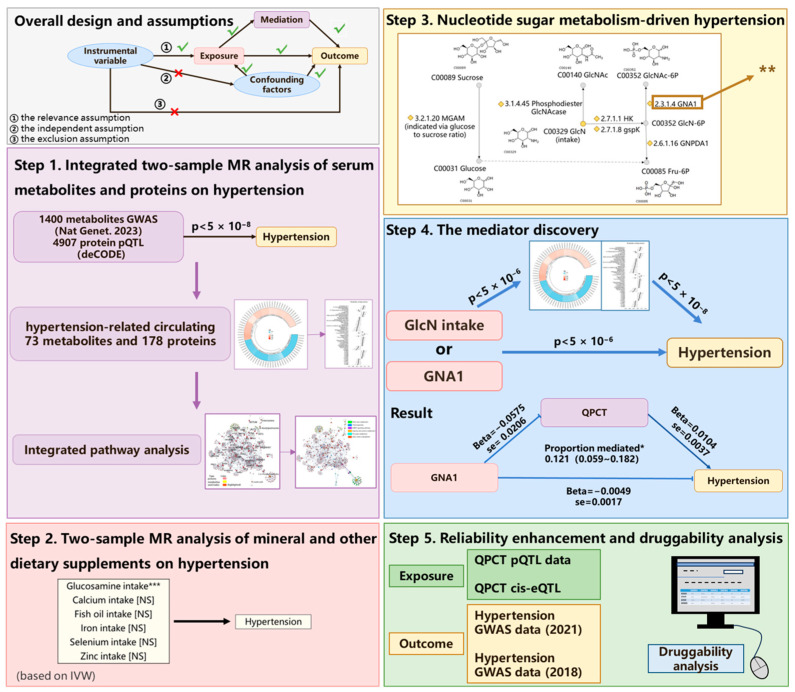
Overview of the study design. Step 1, a two-sample MR study was conducted to assess the relationship between circulating plasma biomolecules and hypertension, using pathway enrichment to explore the underlying mechanisms; Step 2, dietary glucosamine intake was identified as having a negative causal relationship with hypertension; Step 3, GNA1, a key enzyme in nucleotide sugar metabolism, was further investigated in relation to its impact on hypertension; Step 4, two-step MR mediation analysis revealed QPCT as a mediator of the protective effect of GNA1 on hypertension; Step 5, validation was performed using a 2018 GWAS dataset on hypertension and cis-eQTL data for QPCT, with a discussion of QPCT’s druggability for potential hypertension treatments. √, influence exists; ×, not associated; *, *p* < 0.05; **, *p* < 0.01; ***, *p* < 0.001; NS, non-statistical significance.

Figure 1 provides an overview of the workflow. This study aimed to explore the potential associations between dietary supplements and hypertension. It was based on three key assumptions regarding the instrumental variables in MR analysis. Firstly, the relevance assumption requires that the genetic variants should have a measurable influence on the exposure. Secondly, the independent assumption requires the genetic variants to not be associated with any confounding factors that could influence both the risk factor and the outcome. Thirdly, the exclusion assumption states that the genetic variants must affect the outcome solely through the risk factor rather than through alternative pathways [15,29]. In MR studies, the term” exposure” refers to the biological factors or biomarkers that are influenced by genetic variations. These exposures are determined by instrumental genetic variables and are assumed to be randomly allocated at conception, thereby providing a natural experiment to estimate the causal effect on the outcome of interest [15]. In Step 1, the relationship between circulating plasma biomolecules (metabolites and proteins) and hypertension was assessed using a two-sample MR study to explore the potential mechanisms underlying hypertension through pathway enrichment. Subsequently, in Steps 2 and 3, we found that dietary glucosamine intake and glucosamine 6-phosphate N-acetyltransferase (GNA1), a key enzyme in nucleotide sugar metabolism, exhibited a negative causal relationship with hypertension. In Step 4, we conducted a two-step MR design to perform mediation analysis, investigating whether circulating biomolecules could mediate the causal pathway from glucosamine metabolism to hypertension. In this step, glutaminyl-peptide cyclotransferase (QPCT) was identified as a mediator of the protective effect of GNA1 against hypertension. To validate this finding, we utilized another GWAS hypertension dataset from 2018 and the cis-eQTL dataset for QPCT in Step 5. Additionally, we discussed the druggability of QPCT in Step 5 to further enhance the credibility of our findings and to explore potential treatment or preventive strategies for hypertension. This study adhered to the guidelines of the Strengthening the Reporting of Observational Studies in Epidemiology (STROBE), as outlined in Table S1.

### 2.2. Effect of Biomolecule Levels on Hypertension and Integrated Pathway Analysis

Two-sample MR analyses were conducted to examine the relationship between circulating plasma biomolecules, including metabolites and proteins, and hypertension. MR analysis revealed 178 proteins (shown in Table S2) and 73 metabolites (including metabolite ratios, shown in Table S3) causally associated with hypertension. Metabolites provide the phenotype information on hypertension in a close-proximity dimension [32]. Chen et al. identified the associations between metabolite ratios and metabolite-gene expressions [33]. These works provided evidence for integrating metabolite MR results with protein MR results. The pathogenesis of hypertension at the biomolecular level was investigated via these associative biomolecules.

**Figure 2:**
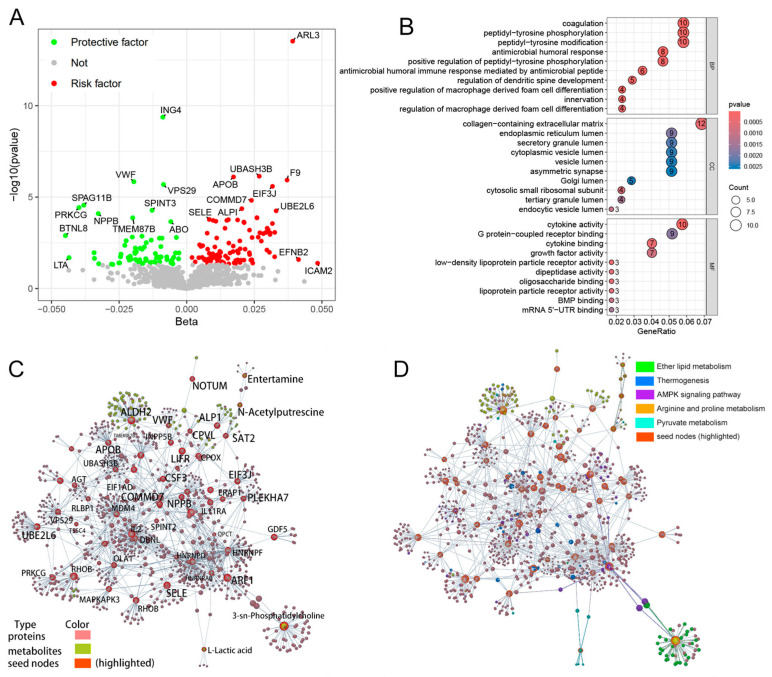
Pathway enrichment of hypertension-associated biomolecules. (**A**). A volcano plot illustrating the proteins that are causally related to hypertension. (**B**). GO enrichment analysis of the proteins with a causal relationship to hypertension. (**C**). A three-dimensional metabolite–protein interaction network generated using OmicsNet. Biomolecules with a causal relationship to hypertension, including metabolites and proteins, were uploaded as seed nodes to investigate the potential pathogenesis of hypertension from a multiomics perspective. Seed nodes in this network are highlighted. (**D**). A three-dimensional metabolite–protein interaction network with significant pathways highlighted to provide a clearer view of the targeted pathways. BP, biological process; CC, cellular component; MF, molecular function.

The GO annotation results based on the 178 proteins causally associated with hypertension shown in Figure 2A revealed that coagulation and peptidyl-tyrosine phosphorylation were predominantly enriched in the biological process (BP) category. Collagen-containing extracellular matrix, endoplasmic reticulum lumen, and secretory granule lumen were identified in the cellular component (CC) category. In terms of molecular function (MF), the results indicated involvement in cytokine activity, G protein-coupled receptor binding, and growth factor receptor binding, as shown in Figure 2B. Figure 2C shows the integrated metabolite–protein interactive network with seed nodes highlighted. The enriched pathways are highlighted to visualize the interactions between nodes in Figure 2D. Notably, the integrated biomolecule pathway enrichment analysis highlighted ether lipid metabolism, thermogenesis, the AMPK signaling pathway, arginine and proline metabolism, and pyruvate metabolism. In this section, the biomolecules that showed a causal relationship with hypertension were identified. In further studies, these biomolecules will be considered as potential mediators.

### 2.3. Impact of Dietary Supplements and Related Exposures on Hypertension

**Figure 3:**
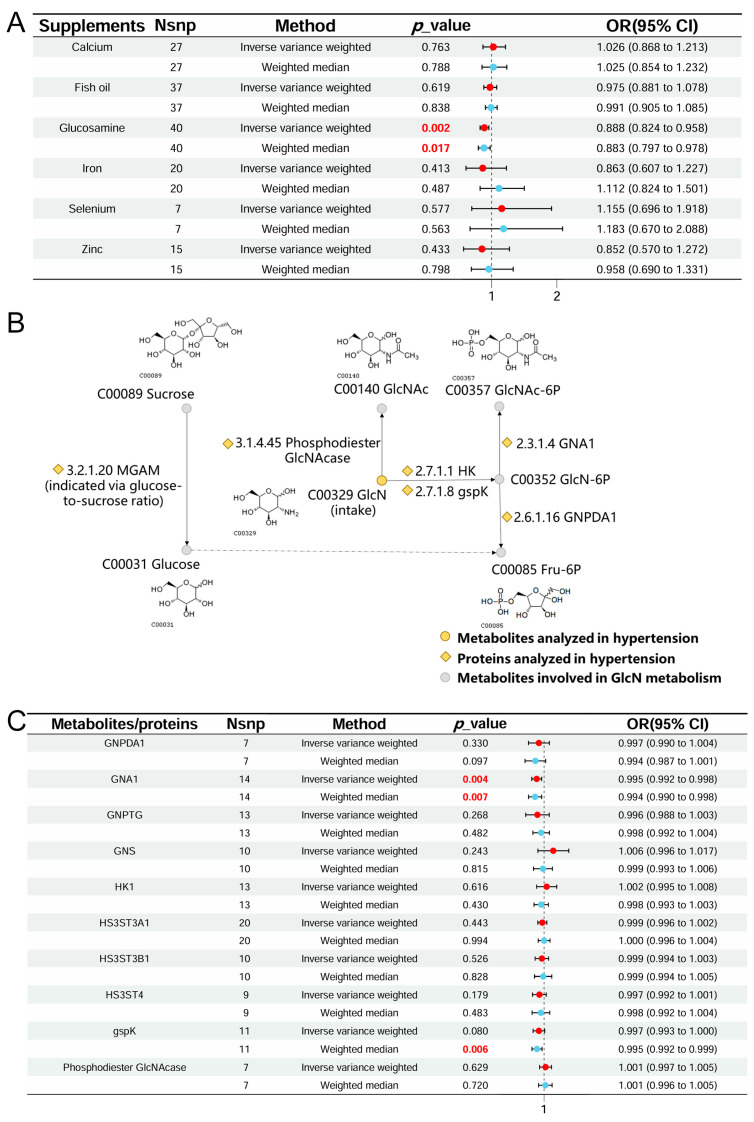
Discovery of hypertension-associated exposure. (**A**). MR estimates (based on IVW) of the effect of dietary supplements on hypertension. (**B**). Amino sugar metabolism pathway and potential exposures. (**C**). MR estimates (based on IVW) of glucosamine metabolism in hypertension. GNPDA1, glucosamine-6-phosphate isomerase 1; GNA1, glucosamine 6-phosphate N-acetyltransferase; GNPTG, N-acetylglucosamine-1-phosphotransferase subunit gamma; GNS, N-acetylglucosamine-6-sulfatase; HK1, hexokinase 1; HS3ST3A1, heparan sulfate glucosamine 3-O-sulfotransferase 3A1; HS3ST3B1, heparan sulfate glucosamine 3-O-sulfotransferase 3B1; HS3ST4, heparan sulfate glucosamine 3-O-sulfotransferase 4; gspK, N-acetyl-D-glucosamine kinase; GlcN, D-glucosamine; GlcN-6P, D-glucosamine 6-phosphate; GlcNAc, N-acetyl-D-glucosamine; GlcNAc-6P, N-acetyl-D-glucosamine 6-phosphate. Exposures with significant causal association with hypertension was highlighted as red fonts.

Healthy dietary patterns have been proven to be helpful in preventing multiple CVDs. The American Heart Association has provided recommendations on dietary patterns, including the Mediterranean diet and the DASH, for preventing CVDs [34]. However, compared to a thorough and strict dietary pattern change, supplement intake is more acceptable to the public. One common feature of these healthy dietary patterns is reducing the intake of components with a high CVD risk and increasing the intake of protective micronutrients such as phytochemicals, unsaturated fatty acids, and minerals [6]. To further discuss which dietary supplements are associated with hypertension, the inverse-variance weighted (IVW) results of dietary supplements on hypertension are shown in Figure 3A and Table S4.

As shown in Figure 3A, glucosamine intake was associated with a reduced risk of hypertension (odds ratio [OR] = 0.888 [0.824–0.958], *p* = 2.00 × 10 ^−3^). To further investigate glucosamine metabolism, a glucosamine metabolism network was constructed in this study, as depicted in Figure 3B. Nodes both upstream and downstream of glucosamine metabolism were considered potential associative exposures in two-sample MR analysis. The IVW results of glucosamine-metabolism-related exposure in hypertension are presented in Figure 3C and Table S5. GNA1 was identified as a protective factor against hypertension (OR = 0.995 [0.992–0.998], *p* = 4.00 × 10 ^−3^). In summary, dietary glucosamine intake and its metabolism through GNA1 were found to have a negative association with hypertension, as demonstrated by MR analysis. This finding provided a basis for further investigation using a two-step mediation MR approach.

### 2.4. Mediation MR Analysis Revealed the Mediative Role of QPCT on Glucosamine-MetaboLlsm-Driven Protection Against Hypertension

**Figure 4:**
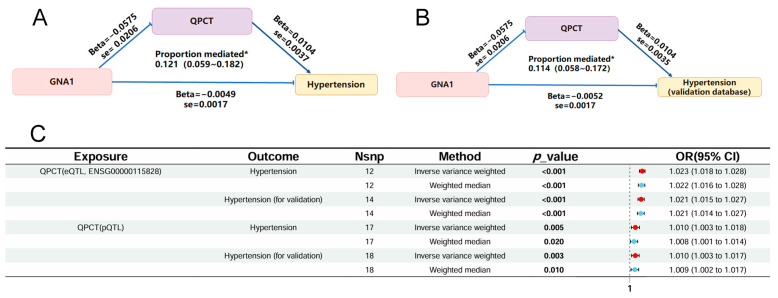
The potential causal evidence summarized and validated from the MR analysis. (**A**). The mediation effects of QPCT on the protective association between GNA1 and hypertension. (**B**). The validation via two-sample MR analysis with the eQTL database and another hypertension GWAS database. (**C**). The identification and validation of the causal relationship between QPCT and hypertension from multiple databases. *, *p* < 0.05.

The plasma metabolites and proteins associated with both glucosamine metabolism and hypertension were identified and measured to determine the mediating effect of each biomolecule. Two-step mediation MR screened out five metabolites and three proteins that were significantly related to both glucosamine intake and hypertension (*p* < 0.05). A total of 11 proteins, 1 metabolite, and 2 metabolite ratios were significantly related to both GNA1 and hypertension (*p* < 0.05). The *p*-value for the mediation effect of each candidate was calculate, as listed in Table S6. After applying the *p*-value filter, cancer-associated plasma membrane protein L2 (CRDL2) and metabolite X-12729 were identified as having a significant mediating effect on glucosamine intake for hypertension protection. Natriuretic peptide B (NPPB), QPCT, and inhibitor of growth 4 (ING4) were also recognized as having a significant mediating effect on glucosamine intake for hypertension protection. However, most of these mediators showed different directions between the direct and indirect effects, which could be attributed to the complexity of the variable relationships. In this study, mediators functioning in different directions were eliminated to ensure mediation consistency. Overall, mediation MR analysis indicated an indirect effect of GNA1 on hypertension protection through QPCT, with a mediated proportion of 12.1% [5.9%–18.2%], as shown in Figure 4A and Table S6. To validate the mediating effect of QPCT, a validation hypertension GWAS dataset was used as exposure. GNA1 showed a protective effect against the validation hypertension dataset (OR = 0.994 [0.992–0.998], *p* = 2.61 × 10 ^−3^). QPCT also showed a significant association with the validation hypertension dataset (OR = 1.010 [1.003–1.017], *p* = 3.44 × 10 ^−3^, shown in Figure 4B), with a mediated proportion of 11.4% [5.8%–17.2%]. The eQTL QPCT database was obtained and screened within ±10 kb of the starting point to assess potential causal evidence and enhance the reliability of the pQTL-GWAS MR result. The eQTL-GWAS result is depicted in Figure 4C, showing a significant association in the eQTL-GWAS MR analysis. There was no evidence of heterogeneity or horizontal pleiotropy among these associations.

### 2.5. Druggability Analysis of QPCT

**Figure 5:**
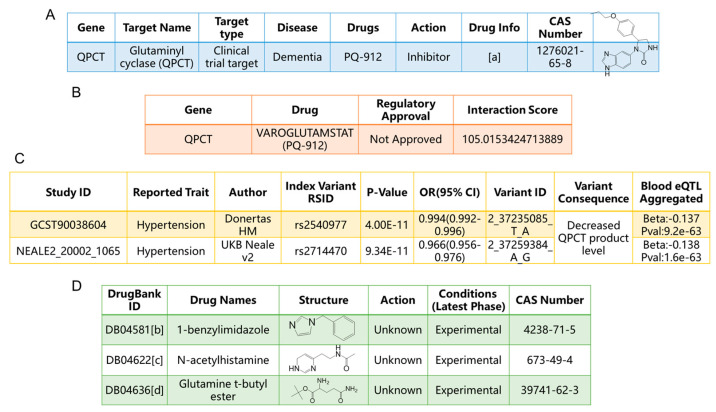
Druggability analysis of QPCT. (**A**). Therapeutic Target Database for QPCT. (**B**). Drug Gene interaction Database for QPCT. (**C**). Open Targets for QPCT. (**D**). DrugBank database for QPCT. Detailed links: [a] https://db.idrblab.net/ttd/data/drug/details/d0ud4y, accessed on 29 June 2024 [35]; [b] https://go.drugbank.com/drugs/DB04581, accessed on 29 June 2024 [36,41]; [c] https://go.drugbank.com/drugs/DB04622, accessed on 29 June 2024 [41]; [d] https://go.drugbank.com/drugs/DB04636, accessed on 29 June 2024 [41].

Our data suggest that glucosamine may be a beneficial supplement for preventing hypertension. This finding raised a new question: could the mediator in this process, QPCT, be a potential drug target for hypertension? To explore the potential application of QPCT as a drug target in hypertension, multiple databases were consulted. The results of the QPCT druggability analysis are depicted in Figure 5. The Therapeutic Target Database identified PQ-912 as an inhibitor of QPCT, a drug–target pair also documented in the Drug Gene Interaction Database, as shown in Figure 5A,B [35,36]. PQ-912 has been demonstrated to inhibit the activity of glutaminyl cyclase and is currently undergoing clinical trials for the treatment of neurodegenerative diseases [37]. DrugBank listed three experimental drugs targeting QPCT: 1-benzylimidazole, N-acetylhistamine, and glutamine t-butyl ester. 1-Benzylimidazole has exhibited potent cardiotonic activity and can inhibit human glutaminyl cyclase [38,39,40,41]. Figure 5C shows that the Open Targets report highlighting the protective role that QPCT inhibitors have played in hypertension studies [42,43]. N-acetylhistamine and glutamine t-butyl ester were both developed as inhibitors of human glutaminyl cyclase, as illustrated in Figure 5D [44,45]. Clearly, the inhibition of QPCT is beneficial for protecting against hypertension. While these drugs have not yet been recognized as QPCT inhibitors for treating hypertension, further research on their potential beyond neurodegenerative diseases is warranted.

## 3. Discussion

Dietary glucosamine intake has been proven to have a significant positive association with a lower risk of all-cause mortality [46]. Clinically, glucosamine has been adopted as a protective supplement for joint pain in patients with osteoarthritis [9]. The anti-inflammatory effect of glucosamine is considered one of the therapeutic mechanisms of glucosamine intake for osteoarthritis [47]. With its mild anti-inflammatory properties, glucosamine is considered safer than other drugs. A total of 2.6% of the worldwide population takes dietary glucosamine supplements [48]. Consequently, studies of the effects of glucosamine and its related metabolic pathways on multiple diseases have been conducted [13,48,49]. Notably, there is sample evidence suggesting that glucosamine might reduce the risk of CVDs. In animal experiments, glucosamine has been shown to improve cardiac function in a resuscitation model after traumatically induced hemorrhage by reducing inflammatory markers [14]. In prospective data analyses, a 7-year follow-up study conducted by the UK Biobank indicated a 15% lower risk of total cardiovascular events caused by glucosamine intake [20]. Zheng et al. conducted another study indicating that glucosamine intake reduces the risk of heart failure by 25% [50]. Consequently, the positive impact of dietary glucosamine intake on cardiovascular diseases has become a focal point for researchers, leading to a significant increase in scholarly attention. However, it remains unknown whether and how glucosamine intake regulates hypertension.

In this study, the negative association between glucosamine intake and hypertension was demonstrated through a two-sample MR study. Our results here are consistent with those of previous works, which demonstrated that the use of glucosamine lowered the risk of CVDs [20,22,46,50]. However, one recent retrospective cohort study reported inconsistent observations, which demonstrated that glucosamine intake was significantly associated with an increased risk of CVDs in patients with osteoarthritis after adjusting for covariates such as hypertension [51]. Considering that this analysis was based on an insurance database, it could not fully capture the potential confounding factors—such as the severity of osteoarthritis, glucosamine consumption, smoking habits, alcohol intake, and exercise intensity [51]. These limitations made it challenging to eliminate the possibility that long-term users of glucosamine, particularly those with osteoarthritis, may already have an increased risk of developing CVDs. The effects of the duration and dosage of glucosamine intake on the occurrence and progression of CVDs in healthy individuals should be determined through more comprehensive clinical studies and animal experiments. Although oral glucosamine intake has been proven to be safe for individuals, several adverse effects including epigastric pain or tenderness, heartburn, diarrhea, nausea, kidney impairment, and even liver toxicity have been reported after glucosamine consumption [52,53,54], indicating that older individuals should carefully consider the potential side effects of glucosamine on their existing illness. Another aspect should be noted is the drug–drug interactions involving glucosamine. Glucosamine may magnify the anticoagulant effect of warfarin, an oral anticoagulant drug, and may reduce the effectiveness of antidiabetic medications [55,56]. Simultaneously taking glucosamine sulfate and acetaminophen may diminish the effectiveness of both supplements and medications [55,57]. These results suggest that patients currently using warfarin, acetaminophen, or antidiabetic medications should be carefully using glucosamine to prevent potential drug interactions. Given these mixed findings, more extensive and well-designed clinical trials, along with animal experiments, are necessary to profile glucosamine’s safety and determine its precise role in the prevention of CVDs, particularly among high-risk populations.

The pathogenesis of hypertension was discussed through integrated protein and metabolite pathway enrichment, as illustrated in Figure 2. Previous studies have indicated that glucosamine intake is linked to these pathways. The downregulated AMPK signaling pathway in the onset of hypertension indicates increased oxidative stress and metabolic disorder [58,59]. Li et al. [60] discovered that glucosamine selectively upregulates *p*-AMPK activity and may help regulate circadian rhythms. These findings suggest that the activation of AMPK pathway underlies the beneficial effect of glucosamine on hypertension development.

To investigate how glucosamine and its metabolism confer protection against hypertension, a genetically predicted mediation study was performed. The results revealed that QPCT may mediate the impact of GNA1 on hypertension (shown in Figure 4A, with a 12.1% proportion mediated) [61]. QPCT catalyzes post-translational chemical reactions on proteins or peptides to convert N-terminal glutamine or glutamate residues into N-terminal pyroglutamate [62,63]. Over the past decades, reducing QPCT activity has mainly been applied to regulate pE-amyloid-β to prevent neurodegenerative diseases [64]. QPCT expression was higher in glioma tumor tissue compared to the adjacent tissue [65]. In recent years, evidence has suggested that QPCT inhibition is effective for myeloid-cell-targeted cancer immunotherapy [66]. Moreover, the beneficial role of the QPCT inhibitor in protecting against hypertension has been proven by multiple public databases [42]. All the data indicated that QPCT may represent a therapeutic target in many cases of pathological dysregulation. Importantly, our work here showed that the QPCT-mediated glucosamine metabolism pathway is involved in the development of hypertension. And thus, the druggability of QPCT as a drug target can inspire further consideration in drug development for hypertension treatment.

Our present study suggests that glucosamine intake plays a protective role in the development of hypertension. Although the potential mechanisms through which glucosamine intake could influence hypertension have not garnered significant interest, some studies have focused on similar treatment approaches for both hypertension and osteoarthritis pain. According to longitudinal studies, individuals with osteoarthritis have a higher likelihood of developing hypertension after adjusting for various confounding factors [67,68]. Statistically, 40 out of 100 patients with osteoarthritis are diagnosed with hypertension [69,70]. Interestingly, hypertension has been recognized as a factor contributing to joint disorders, both biophysically and biochemically [71]. Firstly, fluid flow and intraosseous pressure in local joint tissues are hypothesized to be altered in cases of hypertension, which may impair subchondral bone perfusion [71,72]. Secondly, synovial hypoxia may be triggered by decreased synovial blood flow, leading to cartilage damage [71,73]. Furthermore, hypertension can result in nutrient deprivation for both bone and cartilage, disrupting joint homeostasis [71,74]. In conclusion, research has demonstrated the importance of studying the interplay between osteoarthritis and hypertension. The association between osteoarthritis and hypertension suggests that combination therapy may be effective.

## 4. Materials and Methods

### 4.1. Data Sources

All datasets utilized in this study can be downloaded via publicly available GWASs. Circulating plasma metabolite GWAS datasets were accessed from the recently published human metabolome work from Nature Genetics [33]. This human metabolome work analyzed 1091 plasma metabolite levels and 309 metabolite ratios in 8299 participants aged between 45 and 85 years in the Canadian Longitudinal Study on Aging (CLSA) cohort. The SNP number exceeded 15.4 million.

Circulating plasma protein pQTL datasets were obtained from deCODE Genetics (https://www.decode.com/, accessed on 23 May 2024). DeCODE Genetics used Illumina SNP chips to sequence the whole-genome data with over 27 million SNPs of 49,708 Icelandic-descent individuals. Among these participants, 35,559 individuals participated in the proteomic measurement of 4907 plasma proteins with SOMAscan version 4, a multianalyte, modified aptamer binding assay. The average age of the participants was 55,with a standard deviation of 17 years [75]. The verification of target mediation cis-eQTL data was obtained from the eQTLGen Consortium (https://eqtlgen.org/, accessed on 27 May 2024) [76]. Since the eQTL can reflect the druggability of the target more directly, this work limited the SNPs to within 10 kb upstream of the starting point or 10 kb downstream of the endpoint of our target.

To discuss the mutual causation between dietary supplements (including mineral and other dietary supplements) and hypertension, the genetic instruments for each dietary supplement including glucosamine intake, calcium intake, fish oil intake, iron intake, selenium intake, and zinc intake were obtained from a GWAS dataset in the UK Biobank database (*n* = 461,384). As a longitudinal study, participants in the UK Biobank have undergone long-term follow-up since 2006, when they were aged between 40 and 69. The GWAS datasets for other glucosamine-metabolism-related exposures were obtained from a genomic atlas of the human plasma proteome by Sun et al. The sample size consisted of 3301 blood donors aged 18 years and older from 25 centers within England’s National Health Service Blood and Transplant [77]. The outcome GWAS dataset for hypertension (*n* = 484,598) was obtained from the European Bioinformatics Institute (EBI). The age range of the participants during the first visit was between 37 (minimum age of men = 37, women = 39) to 73 (maximum age of men = 73, women = 71) [42]. The validation GWAS dataset for hypertension (*n* = 461,880) was obtained from the UK Biobank database. A compendium of this data is itemized in Tables S5 and S6. The corresponding datasets can be downloaded from a GWAS catalog (https://www.ebi.ac.uk/gwas/, accessed on 3 May 2024) with the GWAS IDs shown in Table 1. All the data mentioned above were obtained from the relevant literature and public databases, with participant consent and ethical approval.

### 4.2. Instrumental Variable Selection

The instrumental variables in MR should meet the relevance assumption, the independent assumption, and the exclusion assumption for instrumental variables to ensure casual inference, as shown in Figure 1 [15].

In this work, the discovery of associative exposure section included genetic instruments that met the following screening thresholds: (a) significant genome-wide (*p* < 5 × 10 ^−6^) to ensure adequate instrumental variables for discovery, (b) clumped with an LD threshold of r^2^ < 0.001 and a physical distance of less than 10,000 kb to ensure independence, and (c) minor allele frequency (MAF)  >  0.01. Other mediators or exposures in the following steps underwent genetic instrument screening as follows: *p*  <  5 × 10 ^−8^, r^2 ^ <  0.001, kb  <  10,000, and MAF  >  0.01.

### 4.3. MR Analysis and Sensitivity Analysis

As recommended in the guidelines for MR analysis [15], this work applied to the multiplicative random-effects and fixed-effects IVW method as the primary MR. The IVW methods premised that all SNPs functioned as valid instrumental variables under a balanced pleiotropy situation. It amalgamated the Wald ratios of the individual SNPs on the outcomes to perform consolidated casual estimates [78]. For the occurrence of horizonal pleiotropy, pleiotropy-robust MR methods such as the weighted median were implemented to address valid casual estimates [79]. To ensure a precise interpretation of the causal relationship, the Benjamin–Hochberg procedure was employed to calculate the false discovery rate (FDR < 0.05) and avoid false positive results.

Mediation MR analysis was performed with a two-step MR design to investigate whether the level of potential mediators could mediate glucosamine-metabolism-driven protection against hypertension. The two-sample MR effect was calculated in (a) glucosamine-metabolism-related exposures for hypertension, (b) glucosamine-metabolism-related exposures for mediators, and (c) mediators for hypertension. The total effect from the interaction was composed of an indirect effect (effect from couple b*effect from couple c) and a direct effect. The percentage mediated was calculated by dividing the indirect effect by the total effect. The delta approach was applied to generate 95% confidence intervals (CIs) [80]. The Z-value was calculated by dividing the estimated mediation effect by its standard error. The Z-value was used to find the corresponding *p*-value from the standard normal distribution. The *p*-value indicates the probability of observing the mediation effect by chance.

The sensitivity analysis in the MR studies included heterogeneity and horizonal pleiotropy examinations. Heterogeneity among instrumental variables was gauged using Cochran’s Q test. The MR-Egger intercept test [81] and MR Pleiotropy Residual Sum and Outlier (MR-PRESSO) global test [82] were utilized to analyze the potential horizonal and directional pleiotropy. Outliers were eliminated for further MR causal estimations. The impact of each SNP on overall causal estimation was estimated with leave-one-out analysis. The statistical analysis mentioned above was conducted using R software (version 4.3.0). The “Two Sample MR” package was utilized for MR analysis, and the “MR-PRESSO” package was employed for performing the MR-PRESSO analysis.

### 4.4. Metabolite–Protein Interaction Pathway Enrichment and Druggability Analysis

Gene Ontology (GO) and Kyoto Encyclopedia of Genes and Genomes (KEGG) enrichment analysis was performed with the “clusterProfiler” R package to study the pathway enrichment of the associated proteins and their related genes. Three-dimensional views based on the GO and KEGG enrichment results of metabolite–protein interaction networks were constructed with OmicsNet 2.0 (https://www.omicsnet.ca/, accessed 9 June 2024), a web-based tool for visual analysis of biological networks [83,84].

Therapeutic Target Database (update of 5 January 2024, https://db.idrblab.net/ttd/, accessed on 28 June 2024) [36], Drug Gene Interaction Database (version 5.0.3, https://www.dgidb.org/, accessed on 28 June 2024) [35], DrugBank (version 5.1.11, https://go.drugbank.com/, accessed on 28 June 2024) [38], and Open Targets (update of October 2022, https://www.opentargets.org/, accessed on 28 June 2024) [43] were utilized to analyze the druggability of the mediative protein. Potential drugs for hypertension protection via the mediator were discussed in this work.

## 5. Conclusions

In conclusion, this study unveiled the protective role of glucosamine metabolism in hypertension. Dietary glucosamine consumption could prevent the onset of hypertension. Moreover, the ability of glucosamine to protect individuals from the organ damage associated with hypertension, including heart failure, atherosclerosis, and other cardiovascular events, has also garnered attention. The protective influence of glucosamine on hypertension is attributed to its ability to regulate plasma QPCT levels. These results introduce a new therapeutic and preventive approach for hypertension and shed light on the mechanisms of these preventive strategies. Furthermore, our research delved into the multiple biological functions of QPCT, providing fresh perspectives for medication strategies in individuals with hypertension.

## Figures and Tables

**Table 1:** Detailed information about data sources.

Trait	GWAS ID	Year	Sample Size	nSNPs
Glucosamine intake	ukb-b-11535	2018	461,384	9,851,867
Calcium intake	ukb-b-7043	2018	461,384	9,851,867
Fish oil intake	ukb-b-11075	2018	461,384	9,851,867
Iron intake	ukb-b-14863	2018	461,384	9,851,867
Selenium intake	ukb-b-19158	2018	461,384	9,851,867
Zinc intake	ukb-b-13891	2018	461,384	9,851,867
Hypertension	ebi-a-GCST90038604	2021	484,598	9,587,836
Vascular/heart problems diagnosed by doctor: high blood pressure	ukb-b-14177	2018	461,880	9,851,867
GNA1	prot-a-1231	2018	3301	10,534,735
Phosphodiester GlcNAcase	prot-a-1996	2018	3301	10,534,735
HS3ST4	prot-a-1377	2018	3301	10,534,735
HS3ST3B1	prot-a-1376	2018	3301	10,534,735
HS3ST3A1	prot-a-1375	2018	3301	10,534,735
gspK	prot-a-1995	2018	3301	10,534,735
GNS	prot-a-1235	2018	3301	10,534,735
GNPTG	prot-a-1232	2018	3301	10,534,735
GNPDA1	prot-a-1230	2018	3301	10,534,735

## Data Availability

The data presented in this study are available from public databases. The code presented in this study is available upon request from the corresponding author.

## Supplementary Materials

The following supporting information can be downloaded at: https://www.mdpi.com/article/10.3390/ijms252212106/s1.

## List of Abbreviations

**Abbreviation**	**Full Term**
BP	Biological process
CC	Cellular component
CIs	Confidence intervals
cis-eQTL	Cis-expression quantitative trait locus
CLSA	Canadian Longitudinal Study on Aging
CRDL2	Cancer-associated plasma membrane protein L2
CVDs	Cardiovascular diseases
DASH	Dietary Approaches to Stop Hypertension
EBI	European Bioinformatics Institute
FDR	False discovery rate
GlcN	D-glucosamine
GlcN-6P	D-glucosamine 6-phosphate
GlcNAc	N-acetyl-D-glucosamine
GlcNAc-6P	N-acetyl-D-glucosamine 6-phosphate
GNA1	Glucosamine 6-phosphate N-acetyltransferase
GNPDA1	Glucosamine-6-phosphate isomerase1
GO	Gene ontology
GspK	N-acetyl-D-glucosamine kinase
GWASs	Genome-wide association studies
HK	Hexokinase
HS3ST3A1	Heparan sulfate glucosamine 3-O-sulfotransferase 3A1
HS3ST3B1	Heparan sulfate glucosamine 3-O-sulfotransferase 3B1
HS3ST4	Heparan sulfate glucosamine 3-O-sulfotransferase 4
ING4	Inhibitor of growth 4
IVW	Inverse-variance weighted
KEGG	Kyoto Encyclopedia of Genes and Genomes
MAF	Minor allele frequency
MedDiet	Mediterranean diet
MF	Molecular function
MR	Mendelian randomization
MR-PRESSO	Mendelian Randomization Pleiotropy Residual Sum and Outlier
NO	Nitric oxide
NPPB	Natriuretic peptide B
pQTL	Protein quantitative trait loci
QPCT	Glutaminyl-peptide cyclotransferase
SNPs	Single-nucleotide polymorphisms
